# Clinical significance of circulating tumor cells in blood from patients with gastric cancer

**DOI:** 10.1002/ags3.12005

**Published:** 2017-04-25

**Authors:** Takaaki Arigami, Yoshikazu Uenosono, Shigehiro Yanagita, Keishi Okubo, Takashi Kijima, Daisuke Matsushita, Masahiko Amatatsu, Hiroshi Kurahara, Kosei Maemura, Shoji Natsugoe

**Affiliations:** ^1^ Department of Digestive Surgery Breast and Thyroid Surgery Field of Oncology Kagoshima University Graduate School of Medical and Dental Sciences Kagoshima Japan; ^2^ Molecular Frontier Surgery Course of Advanced Therapeutics Kagoshima University Graduate School of Medical and Dental Sciences Kagoshima Japan

**Keywords:** circulating tumor cell, gastric cancer, liquid biopsy, prognosis, tumor progression

## Abstract

Circulating tumor cells (CTC) have been focused on as a target for detecting occult tumors, predicting therapeutic responses and prognoses, and monitoring postoperative recurrence in the clinical management of patients with various malignancies, including gastric cancer. Recent advances in molecular diagnostic tools have contributed to high sensitivity and specificity for the detection of CTC. A conspicuous disparity exists in the incidence of CTC among studies. However, a close relationship has been reported between positivity for CTC and well‐known prognostic clinicopathological factors including depth of tumor invasion, lymph node metastasis, stage, and lymphatic and venous invasion in patients with gastric cancer. According to most studies published on the clinical impact of CTC, the presence of CTC negatively affects the prognosis of patients with gastric cancer. Moreover, the study of CTC based on a meta‐analysis demonstrated their importance as a poor prognostic indicator. In clinical management, pre‐ and post‐therapeutic monitoring of CTC using liquid biopsy may be useful for early detection of subclinical patients or disease recurrence, prediction of tumor progression, and administrative control of adjuvant chemotherapy. Although their functional properties remain unclear, molecular profiling of CTC may contribute to the development of personalized treatment that effectively inhibits tumor progression in patients with advanced gastric cancer. We herein review the clinical significance of CTC as a promising blood marker and therapeutic target in patients with gastric cancer.

## 1 Introduction

Gastric cancer is the fourth most common malignancy in the world and the second leading cause of cancer death.^1^ Advances in diagnostic tools and techniques have resulted in a high incidence of patients with early gastric cancer. Endoscopic resection techniques such as endoscopic mucosal resection (EMR) and endoscopic submucosal dissection (ESD) have been extensively carried out on selected patients with early gastric tumors and free of lymph node metastasis. However, there are clinical limits for accurate tumor detection and diagnoses using preoperative examinations such as endoscopy, endoscopic ultrasonography (EUS), computed tomography (CT), and positron emission tomography‐computed tomography.^2^, ^3^, ^4^ Carcinoembryonic antigen (CEA) and carbohydrate antigen 19‐9 (CA19‐9) are now commonly used as established serum markers in the clinical management of patients with gastric cancer. Nevertheless, the sensitivity and specificity of detecting patients with early gastric cancer are clinically insufficient and few candidate blood markers have clinical utility for overcoming these key problems.^5^

Five‐year survival rates of patients with International Union Against Cancer stages IIIA, IIIB, and IV gastric cancers are 30.8–54.0%, 16.1–36.5%, and 9.2–23.9%, respectively.^6^, ^7^ Furthermore, advances in chemotherapy have contributed to improvements in the prognosis of patients with advanced gastric cancer.^8^ However, difficulties are associated with predicting tumor responses to chemotherapy and disease recurrence after surgery in patients with advanced stage cancer. Although blood monitoring using serum CEA and CA19‐9 has been conventionally introduced for the managements of patients with gastric cancer, serum levels of conventional blood markers do not necessarily coincide with tumor behavior.^9^ Therefore, surrogate blood markers are needed clinically to monitor tumor aggressiveness in real time. Moreover, liquid biopsy using blood specimens has the clinical benefit of being a simple and repeatable sampling tool.

In 1869, the presence of circulating tumor cells (CTC) in peripheral blood was proposed by Ashworth.^10^ CTC are generally isolated from primary tumors or metastatic sites and these cells flow in the bloodstream of patients with malignancies.^11^ To date, CTC have been focused on as a target for detecting occult tumors, predicting therapeutic responses and prognoses, and monitoring postoperative recurrence in the clinical management of patients with various malignancies, including gastric cancer.^12^, ^13^ Non‐invasive liquid biopsy has enabled CTC to be characterized and their numbers assessed. Therefore, the assessment of CTC using liquid biopsy may support new perspectives for the diagnosis and treatment of patients with gastric cancer.

The present review will focus on the clinical significance of CTC as an important therapeutic target in gastric cancer, including recent topics.

## 2 Detection of CTC

Many investigators have reported several approaches for the detection of CTC in patients with gastric cancer. Representative detection methods have been classified into two categories: polymerase chain reaction (PCR)‐based methods and cytometric‐based methods.

Reverse transcription‐polymerase chain reaction (RT‐PCR) is one of the PCR‐based methods. A RT‐PCR assay permits the molecular detection of CTC by assessing the mRNA expression of tumor‐associated markers. Moreover, quantitative RT‐PCR (qRT‐PCR) is a promising tool for quantifying mRNA copy numbers. The greatest advantage of the RT‐PCR assay is its high sensitivity for the molecular detection of CTC. We previously investigated its sensitivity using an *in vitro* model system with serially diluted gastric tumor cells mixed with peripheral blood cells from healthy donors.^14^ The findings of this cell spiking study showed that the RT‐PCR assay detected 10 tumor cells/10^7^ donor‐derived peripheral blood cells. Additionally, a recent RT‐PCR system has the ability to assess multiple gene expressions for the detection of CTC in one run. However, several investigators identified some limitations in the clinical application of RT‐PCR assays to the detection of CTC.^15^, ^16^ False‐positive results associated with RT‐PCR may be yielded as a result of the illegitimate expression of targeted genes by normal cells and epidermal contamination in blood collecting or processing.^15^ Furthermore, false‐negative results may be obtained as a result of the heterogeneous expression of the targeted markers.^16^ Further studies are needed in order to resolve the problems associated with the detection of CTC using RT‐PCR‐based methods.

Table 1 summarizes studies reported since 2001 on CTC assessed using PCR‐based methods in blood specimens from patients with gastric cancer.^16–34^ In RT‐PCR assays, cytokeratin (CK) and CEA are commonly selected as gene target markers for CTC. Both genes are epithelial‐specific antigens that are expressed in the normal cells of gastrointestinal tissues or most tumor cells, including gastric cancer.^35^, ^36^ Recent studies reported the clinical utility of new molecular markers for RT‐PCR assays to detect CTC in the peripheral blood of patients with gastric cancer.^37^ Survivin has been attracting attention as a promising blood marker for CTC in gastric cancer.^25^, ^26^, ^32^ Survivin is a member of the inhibitor of apoptosis gene family and plays an important role in tumor progression.^38^ It has been shown to control tumor apoptosis, promote proliferation, and enhance angiogenesis by a vascular endothelial growth factor signaling pathway.^39^, ^40^ Furthermore, survivin is overexpressed in the tumor cells of various malignant neoplasms, including gastric cancer.^41^ Liu *et al*., in a meta‐analysis of 1365 patients with gastric cancer from 16 eligible studies, demonstrated a close relationship between strong survivin expression in primary tumor sites and a poor prognosis.^42^ Consequently, survivin has potential as an indicator for monitoring CTC in patients with gastric cancer. In contrast, we recently reported the clinical availability of B7‐H3 and B7‐H4 as blood biomarkers of CTC in patients with gastric cancer.^28^, ^31^ These molecules are members of the B7 family and regulate T‐cell‐mediated immune responses.^43^, ^44^ The signaling pathway between B7 family members and their CD28 receptors on activated T cells has a marked impact on the immune surveillance system.^43^, ^44^ Although B7‐H3 is considered to have two opposing characteristics as a co‐inhibitory or co‐stimulatory mediator in T‐cell‐mediated immunity, B7‐H4 is known to function as a negative modulator of immune responses.^43^ Immunohistochemical studies showed that B7‐H3 and B7‐H4 were abundantly expressed in the primary tumor cells of patients with gastric cancer.^45^, ^46^ Accordingly, these immune checkpoint molecules have potential as CTC‐targeted markers to predict tumor responses to chemotherapy and prognoses in the clinical management of patients with gastric cancer. In the near future, the advent of new blood markers is anticipated for the development of an RT‐PCR‐based approach to monitor CTC using liquid biopsy.

**Table 1:** PCR‐based studies on circulating tumor cells in pre‐ and post‐operative blood specimens obtained from patients with gastric cancer

Year	Study	No. patients	Median age, years (range)	UICC stage	Method	Marker	Blood volume for tests (mL)	mRNA levels as the cut‐off value	Patients *vs* Healthy donors		No. patients with CTC (%)	5‐year survival (High/positive *vs* Low/negative)	*P*‐value	Prognostic significance
									No. of healthy donors	Specificity (%)				
2001	Miyazono *et al*.^17^	57	64.4	I–IV	RT‐PCR	CEA	5	–	15	100.0	21 (36.8)	–	–	–
2003	Sumikura *et al*.^18^	106	63.3 (30–87)	–	RT‐PCR	CEA	5	Undescribed	–	100.0	43 (41)	–	–	–
2005	Ikeguchi *et al*.^19^	59	66.3 (26–86)	I–IV	qRT‐PCR	CEA	1.5	–	15	100.0	27 (45.8) ^†^after surgery	–	0.744	No
2005	Illert *et al*.^20^	70	69 (41–87)	I–IV	RT‐PCR	CK‐20	9	–	–	–	28 (40)	42.8% *vs* 74.8%	0.0363	Yes
2005	Seo *et al*.^21^	46	58 (31–78)	I–III	RT‐PCR	CEA	–	–	13	100.0	24 (52.2)	–	–	–
2006	Uen *et al*.^16^	52	60 (34–84)	I–IV	RT‐PCR	c‐Met	4	–	36	94.4	32 (61.5)	–	0.0178	–
2006	Uen *et al*.^16^	52	60 (34–84)	I–IV	RT‐PCR	MUC‐1	4	–	36	91.7	37 (71.2)	–	0.0352	–
2006	Wu *et al*.^22^	64	60.5 (36–84)	I–IV	High‐throughput colorimetric membrane‐array	hTERT	4	–	80	82.5	52 (81.3)	All 4 markers (+) *vs* all 4 markers (−)	0.0223	Yes
2006	Wu *et al*.^22^	64	60.5 (36–84)	I–IV	High‐throughput colorimetric membrane‐array	CK‐19	4	–	80	85.0	50 (78.1)	–	–	–
2006	Wu *et al*.^22^	64	60.5 (36–84)	I–IV	High‐throughput colorimetric membrane‐array	CEA	4	–	80	76.3	53 (82.8)	–	–	–
2006	Wu *et al*.^22^	64	60.5 (36–84)	I–IV	High‐throughput colorimetric membrane‐array	MUC‐1	4	–	80	83.8	54 (84.4)	–	–	–
2008	Koga *et al*.^23^	69	65.9	I–IV	qRT‐PCR	CK‐19	10	103	14	100.0	8 (11.6)	50.0% *vs* 79.0%	0.0347	–
2008	Koga *et al*.^23^	69	65.9	I–IV	qRT‐PCR	CK‐20	10	20	14	100.0	10 (15.5)	51.9% *vs* 78.9%	0.049	–
2008	Mimori *et al*.^24^	810	63.0	I–IV	qRT‐PCR	MT1‐MMP	1	–	29	–	185 (22.8)	–	–	–
2008	Yie *et al*.^25^	55	58 (26–77)	I–IV	qRT‐PCR	Survivin	2	1.07	86	100.0	25 (45.4)	–	0.026	Yes
2009	Bertazza *et al*.^26^	70	68 (28–90)	I–IV	qRT‐PCR	Survivin	6	–	–	–	69 (98.6)	14 mo *vs* 41 mo (median OS)	0.036	Yes
2009	Kita *et al*.^27^	846	61.5 (27–87)	I–IV	qRT‐PCR	uPAR	1	–	25	100.0	404 (47.8)	–	–	–
2010	Arigami *et al*.^28^	94	68 (35–87)	I–IV	qRT‐PCR	B7‐H4	5	0	22	100.0	71 (75.5)	60.4% *vs* 87.2%	0.04	–
2010	Qiu *et al*.^29^	123	59 (28–84)	I–IV	qRT‐PCR	CEA	5	100	30	100.0	45 (36.6)	43.9% *vs* 74.1% (3‐year DFS)	0.001	Yes
2010	Saad *et al*.^30^	30	55 (31–72)	I–IV	qRT‐PCR	CK‐18	2	–	–	–	15 (50.0)	15.2 mo *vs* 35.9 mo (mean DFS)	<0.001	Yes
2011	Arigami *et al*.^31^	95	68 (35–87)	I–IV	qRT‐PCR	B7‐H3	5	–	21	90.0	77 (81)	57.1% *vs* 76.4%	0.02	Yes
2011	Cao *et al*.^32^	98	–	I–IV	qRT‐PCR	Survivin	6	1.25	30	100.0	45 (45.9)	84.3% *vs* 53.1% (3‐year DFS)	<0.001	Yes
2013	Arigami *et al*.^33^	93	68 (35–87)	I–IV	qRT‐PCR	STC‐2	5	–	22	–	43 (46.2)	58.4% *vs* 80.9%	0.014	–
2013	Kang *et al*.^34^	118	–	I‐IV	qRT‐PCR	hTERT	6	0.18	58	87.0	66.0	33.4% *vs* 54.2%	<0.001	Yes

CEA, carcinoembryonic antigen; CK, cytokeratin; c‐Met, hepatocyte growth factor receptor; CTC, circulating tumor cells; DFS, disease‐free survival; hTERT, human telomerase reverse transcriptase; mo, months; MT1‐MMP, membrane‐type‐1 matrix metalloproteinase; MUC, mucin; OS, overall survival; qRT‐PCR, quantitative reverse‐transcription–polymerase chain reaction; RT‐PCR, reverse transcription–polymerase chain reaction; STC, stanniocalcin; UICC, International Union Against Cancer; uPAR, urokinase‐type plasminogen activator receptor; –, undescribed.

Table 2 summarizes studies reported since 2007 on CTC assessed by cytometric‐based methods using blood specimens from patients with gastric cancer.^47–55^ The CellSearch system (Janssen Diagnostics, Raritan, NJ, USA) is one of the representative CTC detection assays using a cytometric‐based method. This system has been approved by the American Food and Drug Administration (FDA) as a diagnostic tool for detecting CTC in patients with metastatic breast, colorectal, and prostate cancer.^56^ In the CellSearch system, CTC are captured based on enrichment using antibody‐coated magnetic beads with epithelial‐cell adhesion molecules and discrimination using fluorescently labeled antibodies against CK and CD45. We investigated the presence or absence of CTC in peripheral blood cells from patients with gastric cancer using the CellSearch system.^51^ The findings obtained showed that CTC were morphologically detected using the CellSearch system, particularly in patients with unresectable advanced or recurrent gastric cancers. Recently, a new size‐based separation system has been developed for enrichment and cultivation of CTC.^55^ The greatest appeal of this system is that it can easily separate viable CTC from peripheral blood. Moreover, we can assess functional properties by culture of enriched viable CTC. Accordingly, the size‐based filtration system may be focused as a novel tool for isolating viable CTC.

**Table 2:** Cytometric‐based studies on circulating tumor cells in blood specimens obtained from patients with gastric cancer

Year	Study	No. patients	Mean age, years (range)	UICC stage	Method		Marker	Blood volume for tests (mL)	No. CTC as the cut‐off value	Patients *vs* Healthy donors		No. patients with CTC (%)	5‐year survival (High/positive *vs* Low/negative)	*P*‐value	Prognostic significance
					Enrichment	Detection				No. healthy donors	Specificity (%)				
2007	Pituch‐Noworolska *et al*.^47^	57	55.0	I–IV	FACS	ICC	CK‐8, CK‐18, CK‐19	Undescribed	≥3	–	–	31 (54.4%)	–	≥0.05	No
2008	Hiraiwa *et al*.^48^	27	–	IV	CellSearch	ICC	EpCAM, CK‐8, CK‐18, CK‐19	7.5	≥2	41	100	15 (55.6%)	–	0.039	Yes
2010	Matsusaka *et al*.^49^	52	62 (24–78)	IV	CellSearch	ICC	EpCAM, CK‐8, CK‐18, CK‐19	7.5	≥4	–	–	17 (32.7%)	3.5 mo *vs* 11.7 mo (median OS)	<0.001	Yes
2012	Ito *et al*.^50^	65	58.8 (33–76)	I–IV	TelomeScan (GFP)	ICC	EpCAM	7.5	≥5	–	–	–	–	0.0021	–
2013	Uenosono *et al*.^51^	148	–	I–IV	CellSearch	ICC	EpCAM, CK‐8, CK‐18, CK‐19	7.5	≥1	15	100	16 (10.8%)	–	<0.0001	Yes
2013	Uenosono *et al*.^51^	103	–	IV	CellSearch	ICC	EpCAM, CK‐8, CK‐18, CK‐19	7.5	≥1	15	100	62 (60.2%)	248 days *vs* 582 days (median OS)	0.0044	
2015	Li *et al*.^52^	44	56 (25–87)	I–IV	CanPatrol	RNA‐ISH	KRT‐8, KRT‐18, KRT‐19, EpCAM	5	≥1	10	100	35 (79.5%)	–	–	–
2015	Okabe *et al*.^53^	136	66.0	I–IV	CellSearch	ICC	EpCAM, CK‐8, CK‐18, CK‐19	7.5	≥1	–	–	25 (18.4%)	–	0.016	Yes
2015	Yuan *et al*.^54^	31	62 (35–78)	II–IV	FACS	ICC	CD44	2	–	–	–	14 (45.2%)	–	–	–
2016	Kolostova *et al*.^55^	22	–	I–IV	MetaCell	ICC	CK‐18, CK‐19, CK‐20, CK‐7, EpCAM, MUC‐1, HER‐2, EGFR	8	–	–	–	13 (59%)	–	–	–

CanPatrol, SurExam, Guangzhou, China; CellSearch, Janssen Diagnostics, Raritan, NJ, USA; MetaCell, MetaCell s.r.o., Ostrava, Czech Republic; TelomeScan, Oncolys BioPharma Inc., Tokyo, Japan.

CK, cytokeratin; CTC, circulating tumor cells; EGFR, epidermal growth factor receptor; EpCAM, epithelial cell adhesion molecule; FACS, fluorescence‐activated cell sorting; GFP, green fluorescent protein; HER, human epidermal growth factor receptor; ICC, immunocytochemistry; ISH, *in situ* hybridization; KRT, keratin; mo, months; MUC, mucin; OS, overall survival; UICC, International Union Against Cancer; –, undescribed.

## 3 Incidence of CTC

The incidence of CTC ranges between 11.6% and 98.6% in studies based on PCR‐based methods (Table 1).^16‐34^ The gap observed in the incidence of CTC among each study may be as a result of differences in the clinicopathological backgrounds of enrolled patients, target markers, blood volumes assessed by PCR, and the cut‐off values for mRNA levels. However, the incidence of CTC ranged between 36.6% and 52.2% in five RT‐PCR studies targeting CEA, which is one of the conventional PCR markers for the detection of CTC.^17^, ^18^, ^19^, ^21^, ^29^ According to studies assessed in this review article, positive rates of serum CEA ranged between 24.3% and 26.3%.^21^, ^29^, ^31^ These results suggest that sensitivity of CEA mRNA levels is higher than those of serum CEA levels. Furthermore, in a large‐scale study on 846 patients with stages I–IV gastric cancer, Kita *et al*.^27^ reported that positivity for CTC using a qRT‐PCR assay with the urokinase‐type plasminogen activator receptor was 47.8% (404/846). In contrast, specificity ranged between 76.3% and 100% in these studies.^16–19,21–23,25,27–29,31,32,34^ These findings indicate that PCR‐based methods have clinical availability for discriminating between healthy donors and patients with gastric cancer.

In a study on 57 patients with stages I–IV gastric cancer, Miyazono *et al*. reported that the positive rates for CEA mRNA expression before and after gastrectomy were 8.8% (5/57) and 33.3% (19/57), respectively.^17^ Moreover, they demonstrated a close relationship between the presence or absence of CEA mRNA expression and disease recurrence, such as liver metastases.^17^ The findings of this study suggest that surgical maneuvers enhance the metastatic process from the detachment of primary tumor cells into the systemic circulation in patients with gastric cancer. Therefore, sequential evaluations based on pre‐ and postoperative PCR‐based assays are anticipated to monitor disease recurrence in patients with gastric cancer.

According to studies based on cytometric‐based methods, the incidence of CTC ranges between 10.8% and 79.5% (Table 2).^47–55^ The CellSearch system was previously used to detect CTC in four (44.4%) out of nine studies using cytometric‐based assays. The findings of these CellSearch studies demonstrated that the incidence of CTC ranged between 10.8% and 18.4% and between 32.7% and 60.2% in patients with stages I–IV and stage IV, respectively.^48^, ^49^, ^51^, ^53^ These findings indicate that incidence of CTC is higher in patients with than in those without distant metastasis.

The incidences of CTC as determined by PCR assay and cytometric‐based methods in patients with stage I were 12.5–58.3% and 27.5–69.2%, respectively.^16,17,20,22,24,25,29,47,50,51,55^ These results suggest that patients with early tumors tend to display a low incidence of CTC compared with those with advanced tumors. Consequently, the clinical significance of CTC in early gastric cancer remains controversial at present. However, according to a systematic review, positive rates of serum CEA and CA 19‐9 in patients with stage I were 13.7% and 9.0%, respectively.^5^ These findings indicate that PCR‐based or cytometric‐based tools for CTC have a high sensitivity for detecting early tumors in comparison with conventional serum CEA or CA 19‐9 markers. These abilities will assist clinical management in patients with early gastric cancer.

## 4 Relationship between clinicopathological factors and CTC

To date, many investigators have reported a close relationship between positivity for CTC and well‐known prognostic factors, such as tumor size, depth of tumor invasion, lymph node metastasis, stage, lymphatic and venous invasion.^16–34,47–55^ In a study on 94 patients with gastric cancer, we reported that the presence or absence of CTC evaluated by a qRT‐PCR assay for the expression of B7‐H4 correlated with the depth of tumor invasion, lymph node metastasis, stage, lymphatic invasion, and venous invasion (*P *= 0.006, *P *= 0.001, *P *< 0.001, *P *< 0.001, and *P *= 0.01, respectively).^28^ In a study on 148 gastric cancer patients receiving surgical treatment, Uenosono *et al*.^51^ reported that CTC assessed by the CellSearch system correlated with the depth of tumor invasion, lymph node metastasis, distant metastasis, stage, lymphatic invasion, and venous invasion (*P *= 0.009, *P *< 0.0001, *P *= 0.012, *P *= 0.0002, *P *= 0.0003, and *P *= 0.006, respectively). These findings suggest that blood assessments for the detection of CTC have the clinical power to predict tumor progression and malignant aggressiveness in patients with gastric cancer.

## 5 Prognostic impact of CTC

A large number of studies have investigated the clinical significance of CTC in patients with various malignancies, such as esophageal cancer, colorectal cancer, and pancreatic cancer.^57^, ^58^, ^59^ Similarly, many investigators have assessed the prognostic impact of CTC in patients with gastric cancer, and most studies have suggested a close relationship between the presence of CTC and a poor prognosis.^16–34,47–55^

In a qRT‐PCR study of 123 gastric cancer patients with stages I–IV, Qiu *et al*.^29^ reported that 3‐year disease‐free survival (DFS) rates in patients who were positive or negative for CEA mRNA were 43.9% and 74.1%, respectively (*P *= 0.001). A multivariate analysis identified CEA mRNA positivity as an independent prognostic factor (*P *= 0.02).^29^ Moreover, the sensitivity and specificity of CEA mRNA expression for predicting disease recurrence were 56.8% and 74.7%, respectively.^29^ However, the sensitivity and specificity of the serum CEA status were 31.8% and 79.7%, respectively.^29^ They concluded that the presence or absence of CTC by qRT‐PCR detection for CEA mRNA was a promising predictor for disease recurrence in patients with gastric cancer.^29^ In contrast, in a qRT‐PCR study on 59 gastric cancer patients with stages I–IV, Ikeguchi and Kaibara reported that there were no significant differences in overall survival (OS) rates among patients with or without CEA mRNA expression (*P *= 0.744).^19^ In that study, CTC were assessed using a qRT‐PCR assay on blood specimens after gastrectomy.^19^ The findings obtained indicated that CTC were destroyed shortly after gastrectomy.^19^ They hypothesized host‐related immunological defense mechanisms as one of the reasons for these findings.^19^

Cao *et al*.^32^ focused on survivin as a novel blood marker of CTC in a qRT‐PCR study on 98 gastric cancer patients with stages I–IV.^32^ They reported that 3‐year DFS rates in patients who were positive or negative for survivin mRNA were 53.1% and 84.3%, respectively (*P *< 0.001). Furthermore, a multivariate analysis identified the status of survivin mRNA as an independent prognostic factor (*P *< 0.001).^32^ In a study on 55 gastric cancer patients with stages I–IV, Yie *et al*. showed that the specificity, sensitivity, and accuracy of survivin‐expressing CTC for predicting disease recurrence were 100%, 100%, and 84.6%, respectively.^25^ Bertazza *et al*.^26^ compared survivin with other blood markers, such as CEA, CK‐19, and vascular endothelial growth factor (VEGF), in order to select the most suitable mRNA marker for predicting clinical outcomes in a qRT‐PCR study on 70 gastric cancer patients with stages I–IV. Univariate and multivariate analyses identified only the status of survivin mRNA expression as an independent prognostic factor.^26^ These studies suggest that qRT‐PCR assays for survivin expression support the planning of strategic treatment, particularly in patients with advanced gastric cancer who occasionally develop disease recurrence.

In recent years, immunotherapy has begun to attract attention as a drug treatment for patients with several malignant neoplasms.^60^ According to the findings of a phase 1b trial on immunotherapy for patients with advanced gastric cancer, the anti‐programmed cell death protein 1 (PD‐1) antibody pembrolizumab was found to be safe and exerted antitumor effects.^61^ Although PD‐1 is one of the representative molecules for immune checkpoints, we focused on other immune checkpoint molecules, such as B7‐H3 and B7‐H4.^28^, ^31^ We investigated the prognostic impact of B7‐H3 and B7‐H4 in the peripheral blood of patients with stages I–IV gastric cancer.^28^, ^31^ In a qRT‐PCR‐based study on 95 patients with gastric cancer, 5‐year OS rates in patients who strongly or weakly expressed B7‐H3 were 57.1% and 76.4%, respectively (*P *= 0.02).^31^ Additionally, multivariate analyses selected the status of B7‐H3 expression as an independent prognostic factor (*P *= 0.046).^31^ In a B7‐H4 study on 94 patients with gastric cancer, 5‐year OS rates in patients who were positive or negative for mRNA expression were 60.4% and 87.2%, respectively (*P *= 0.04).^28^ Our findings propose that the evaluation of B7‐H3 and B7‐H4 mRNA expression in blood specimens is useful as a CTC‐associated tool for predicting the prognosis of patients with gastric cancer.

In a CellSearch study on 136 gastric cancer patients with stages I–IV, Okabe *et al*.^53^ reported that progression‐free survival was significantly shorter in patients with than in those without CTC (*P *= 0.016). All other studies based on the CellSearch system demonstrated that CTC had an influence on prognosis.^48^, ^49^, ^51^ These findings suggest that the presence or absence of CTC has an effect on the prognosis of patients with gastric cancer, even in cytometric‐based methods.

In a meta‐analysis on 19 studies regarding CTC from patients with gastric cancer, Wang *et al*. reported that positivity for CTC correlated with poor OS (HR: 2.42, 95% CI: 1.94–3.02, *P *< 0.001).^62^ Although further clinical studies including a molecular analysis of CTC are needed to reach definitive conclusions on this matter, it is highly likely that CTC negatively affect the prognosis of patients with gastric cancer.

## 6 Future perspectives for CTC as a promising blood marker and therapeutic target

The advent of new blood markers for the detection of CTC is anticipated in the clinical management of patients with gastric cancer. Potential markers for CTC may be clinically identified in the near future and, thus, it may become possible to discriminate subclinical patients with early tumors and accurately predict tumor progression and prognosis in patients with gastric cancer. Additionally, the assessment of CTC in blood may improve the selection of patients for neoadjuvant systemic chemotherapy in pre‐therapeutic management of gastric cancer. Moreover, the postoperative monitoring of CTC by liquid biopsy may be useful for the early detection of disease recurrence and the administrative control of adjuvant chemotherapy. As technologies have been developed for the isolation and enrichment of tumor cells, we will be able to easily isolate viable CTC from blood specimens using promising markers.

The functional properties of CTC currently remain unclear. The main reason for this is that *ex vivo* cultures of CTC represent a challenging approach in clinical experiments. However, recent advances in basic research have contributed to elucidating CTC‐associated biological behaviors in patients with several malignancies. Yamamoto *et al*.^63^ developed a novel method for the *ex vivo* culturing of CTC using a fibroblast feeder layer and magnetic coculture protocol. They cultured CTC isolated from the blood specimens of metastatic mouse models and obtain three CTC‐derived cell lines.^63^ They demonstrated that the malignant behavior of CTC‐derived cell lines were more aggressive than that of the original cells.^63^ Furthermore, Alix‐Panabières *et al*.^64^ reviewed functional studies on CTC using *in vitro* cultures and *in vivo* xenograft models. They concluded that CTC‐derived cell lines and xenograft models are promising tools for examining the molecular properties of CTC and identifying new therapeutic targets.^64^

Several researchers have proposed a close relation between CTC and cancer stem cell‐like properties or epithelial mesenchymal transition in various malignancies, including gastric cancer.^54^, ^56^ Further understanding of their relationship might allow the progress of a new CTC‐targeted therapy that controls hematogenous metastasis in patients with gastric cancer. Accordingly, the molecular profiling of CTC by liquid biopsy will contribute to the development of personalized treatment that effectively inhibits tumor progression in patients with advanced gastric cancer.

## Conflicts of Interest

Authors declare no conflicts of interest for this article.
